# Developing tools for defining and establishing pathways of toxicity

**DOI:** 10.1007/s00204-015-1512-y

**Published:** 2015-04-08

**Authors:** Melvin E. Andersen, Patrick D. McMullen, Daniel Krewski

**Affiliations:** 1 The Hamner Institutes for Health Sciences, Research Triangle Park, NC 27709-2183 USA; 2 McLaughlin Centre for Population Health Risk Assessment, University of Ottawa, Ottawa, ON Canada; 3 Risk Sciences International, Ottawa, ON Canada

**New approaches for toxicity testing**: The US National Research Council report on ‘Toxicity Testing in the 21st Century’ (Krewski et al. 2010) envisioned a shift in testing away from studies of apical endpoints in test animals to the use of human cells to assess perturbations of toxicity pathways (TPs). The report generated widespread interest and has produced subsequent discussions regarding implementation of its key recommendations (Andersen and Krewski 2010; Krewski et al. 2011, 2014). TPs were defined as normal cellular signaling pathways that could serve as targets of toxicity in the face of perturbations of their function by chemical exposures. The examples provided in the report included sex steroid hormone receptor pathways, liver nuclear receptor signaling, and the suite of eight canonical stress pathways, including oxidative stress, DNA damage, heat shock, hypoxia, metal stress, inflammation, endoplasmic reticulum stress, and oxidative stress (Simmons et al. 2009). This aggregation of pathways, based largely on preexisting biological information, remains coarse-grained with many possible nodes in each of these TPs whose alterations could lead to toxicity. Some of the continuing challenges in advancing new, cell-based methods for toxicity testing are (1) the manner in which testing will be accomplished, (2) the degree of detail required to define the biological targets whose alterations lead to toxicity, and (3) the biological granularity underpinning definitions of toxicity pathways.

The path forward clearly requires use of multiple approaches for identifying targets of toxicity and the methods for querying how biological perturbations of these targets lead to toxic responses. Several recent initiatives show different approaches for using pathway information in safety assessments and developing a clear vocabulary regarding these ‘pathways.’ The ToxCast™ screening initiative at the US EPA assesses the most sensitive molecular initiating event (MIE) and uses multiple readouts from in vitro assays to assess potency for the various endpoints expressed (Judson et al. 2010; Sipes et al. 2013). The concentrations causing changes in biological response are compared to the human exposure levels necessary to give circulating concentrations in a person equivalent to those active in the in vitro assays (Wetmore et al. 2012). This comparison—which effectively represents a ‘margin of exposure’—can assist in prioritizing compounds for further testing (either in vitro or in conventional animal testing) (Thomas et al. 2013). Case study advocates (Andersen et al. 2011) use existing knowledge of chemicals with specific modes of action to develop the tools for improving assay design and for conducting in vitro–in vivo pharmacokinetic and low-dose pharmacodynamic extrapolations (Adeleye et al. 2014). The high-throughput screening and case study approaches rely on preexisting knowledge to develop assays, design readouts, and propose interpretive tools for use of the information in human safety assessments. In addition to looking at chemicals or chemical libraries where there is significant preexisting knowledge about MIEs, other assays that are agnostic with respect to pathway targets and provide a breadth of information to infer either targets or infer safe exposures are needed. New bioinformatic tools will need to take output from these assays and generate information on the networks altered by exposure and the affected pathways. The Human Toxome Project proposes to apply unbiased multiomic tools to map pathways of toxicity (PoTs) in human cells (Hartung and McBride 2011).

**Advancing toxicity test information content through the development of PoTs**: A continuing question relates to the level of detail to be included in defining PoTs and the bioinformatic and computational tools required in creating both static and dynamic representations of PoTs. High-content data streams, such as gene expression microarrays, chromatin immunoprecipitation (ChIP), and metabolomics, are now well-established tools for molecular biology. In their application, output from these technologies need to be integrated to provide more than simply lists of features (such as genes, proteins, and metabolites) altered following treatment of cells with or exposures of test animals to particular chemicals. Their integration promises insights into the molecular targets, MIEs and the nature of the downstream processes whose perturbation lead to toxicity. The PoT concept encompasses the detailed steps by which cells sense toxic stressors and then respond to these perturbations. The PoT describes in more detail the cellular machinery recruited either by activation of cellular stress pathways or by direct activation of cellular signaling through receptor-mediated pathways. Gene expression changes, altered metabolite concentrations, or other biochemical markers are measureable indicators of cellular and biochemical changes. Relating these changes to conventional adverse endpoints and defining adversity (Boekelheide and Andersen 2010) at the cellular level are necessary elements within PoT framework.

In this issue of Archives of Toxicology, Maertens et al. (Maertens et al. 2015) take the first step in developing a PoT for adverse response to MPTP, a compound that causes neuropathy similar to Parkinson’s disease. MPTP affects dopaminergic neurons in the substantia nigra through several processes, including mitochondrial dysfunction and microtubule disruption. However, the signaling network and the specifics of the cascade of responses initiated by MPTP remain only vaguely understood. Due to its similarity to parkinsonian disorders and the examination of cases of human toxicity of MPTP, this compound has been extensively studied, making it an excellent candidate for the development of a compound-specific PoT.

**Leveraging existing data sets for developing pathways of toxicity**: High-content data streams can provide hypothesis-free snapshots of changes to cellular state in response to toxic insults. Maertens et al. (2015) show how to use existing gene expression and transcription factor (TF) data sets to build PoTs de novo. In this case, the PoT reflects the TF network activated in response to MPTP exposure in mice, along with the genes transcribed as a consequence. To begin the examination of the PoT, Maertens et al. access a published data set on whole genome gene expression results from the substantia nigra of male B6C3F1 mice dosed at 10 weeks of age for 3 days with either 30 mg MPTP/kg or saline control and killed either 24 h or 7 days after the final dose (Miller et al. 2004). Weighted gene-correlation network analysis extracted an initial network from the suite of differentially expressed genes, identifying five statistically significant modules. The authors use this published study to define transcriptional changes and combine these results with text-mining techniques and gene regulatory information from various databases to refine the description of the PoT. Through the application of these other tools, the authors identified key nodes within the overall network and the connections between transcription factors and groups of genes. The most densely connected transcription factor was SP1, whose products appear to play multiple roles in the cellular responses to MPTP.

Clearly, the pathway/network analysis in their paper is preliminary in nature due to the limited data set used for the analysis. However, the importance of the paper relates more to the use of tools to unravel the sequence of cellular alterations and the interacting modules that contribute to the altered cellular phenotype following MPTP treatment. Future applications could assess the manner in which these network biology tools might provide information about targets and pathways using a consistent workflow designed to uncover PoTs from other high data content studies. Over time, these network analyses will be enriched by the use of broader data sets (for multiple doses and times of treatment) and from the use of multiple data streams—e.g., ChIP-seq and metabolomics. Identifying gene regulatory networks—the set of transcription factors and the genes they regulate via cis interactions—has been an active area of investigation for over a decade (Bar-Joseph et al. 2003; Friedman 2004; Segal et al. 2003). Today the methods available for these analyses are moving forward quickly. Rapid advances in high-throughput ChIP have breathed new life into the problem of mapping gene regulatory networks based on gene expression microarray data. It will be interesting to see these expanded tool sets brought to bear more regularly in both identifying perturbations that become associated with PoTs and evaluating the specific criteria for defining the PoT concept and creating a PoT ontology.

One of the major advantages of using high-content data streams for pathway mapping is that these techniques are intrinsically untargeted. The biochemistry of many PoTs, for example those involving the activation of nuclear hormone receptors, are at least partially understood. Nuclear receptors are bound by ligands—either native or exogenous—and drive transcriptional programs by directly binding regulatory elements of target genes. However, this coarse-grained description of the pathway does not address important toxicological issues, such as the relevance to human health of tumors formed in rodents by sustained activation of PPARα, CAR, and AhR. Furthermore, most genes transcribed in response to nuclear receptor activation are not directly bound by the nuclear receptor itself (Dere et al. 2011; McMullen et al. 2014). High-throughput data sets, because they do not rely on prior assumptions about the chemical’s mode of action, can identify the non-canonical elements of these PoTs and provide more detail into the nature of the interactions leading to alterations of the normal biological signaling networks.

**Some thoughts on pathways, modes of action and ‘pathways of toxicity’**: Based on directions within the ToxCast™ and Tox21 (Attene-Ramos et al. 2015) communities in the USA, it is likely that the coming years should see increasing emphasis on evaluating adverse cellular responses using in vitro transcriptomic studies in a group of human cells/cell lines. These studies would evaluate multiple doses across a limited number of time points. The data from these assays should change the landscape for assessing toxicity by assessing dose–response using benchmark dose (BMD) or another statistical analyses for changes in gene expression (Thomas et al. 2007; Yang et al. 2007; Sand et al. 2011). Here, the targets would be defined as enriched ‘pathways’ from various ontology platforms such as MetaCore, Reactome, and Gene Ontology—from which BMDs are estimated. The simplest readouts assess the most sensitive enriched units—the enriched pathway with the lowest BMD. This readout is similar to assessing the most sensitive endpoint across multiple apical studies by finding the endpoint with the lowest BMD (or other point of departure). Unfortunately, this readout is largely uninformative for assessing PoTs. Clearly, analyses that stop at reporting the lowest BMD for specific pathways will not really take advantage of the emerging biological information accessible from the patterns and dose–response behaviors in these data sets. The PoT tools from the Maertens et al. study can be organized into a pipeline for analysis of more detailed gene expression studies and contribute to defining PoTs and, in the longer term, to creating a more detailed ontology of PoT for the diverse compounds in the ToxCast™ library. Progress in defining PoTs for larger numbers of compounds could also assist in creating ontologies of human PoTs. The emerging application of AOPs (Vinken 2013) also could make use of PoTs, since PoTs serve as the link between MIEs and specific in vitro assays to assist regulatory decision making in both North America and the EU. This paper by Maertens and colleagues provides a glimpse of emerging methods to extract PoTs from multiomic data streams and, indirectly at least, allows us to think about how these PoTs will assist assay design and application of this information in new directions in chemical risk/safety assessment.
